# Longitudinal Changes in Glutamine and Ammonia in Relation to Hyperammonemic Crisis in Urea Cycle Disorders

**DOI:** 10.1002/jmd2.70105

**Published:** 2026-06-18

**Authors:** Yasuaki Yasuda, Yoko Nakajima, Yuta Sudo, Takuma Ishihara, Tetsuya Ito

**Affiliations:** ^1^ Department of Pediatrics Fujita Health University School of Medicine Toyoake Japan; ^2^ Innovative and Clinical Research Promotion Center Gifu University Hospital Gifu Japan

**Keywords:** ammonia, biomarker, glutamine, hyperammonemia, neonatal‐onset, urea cycle disorders

## Abstract

Hyperammonemic crisis (HAC) remains a major risk factor for urea cycle disorders (UCD), and practical outpatient predictors are limited. We tested whether short‐term changes in plasma glutamine (ΔGln) and ammonia (ΔNH_3_) predict HAC and whether effects differ by onset type. In a retrospective cohort (2014–2024) of 18 patients with UCD (neonatal‐onset [NO] nine; late‐onset [LO] nine), HAC was defined as ammonia (NH_3_) > 150 μg/dL (88.1 μmol/L). For each patient, ΔGln and ΔNH_3_ were calculated between sequential outpatient samples. Investigation 1 compared the changes observed between 31–60 days and 8–30 days before HAC, with stable period changes. Investigation 2 compared changes at 61–90 and 31–60 days before HAC with stable period changes. Associations were evaluated using generalized linear mixed‐effects models with onset‐specific effects. In NO, larger ΔGln during Investigation 1 was associated with higher HAC risk (*p* < 0.001) whereas ΔNH_3_ was not associated with HAC (*p* = 0.361). The probability of HAC in NO was estimated to reach 67.1% at ΔGln +500 μmol/L. In LO, neither ΔGln nor ΔNH_3_ during Investigation 1 showed a significant association with HAC, and the estimated probabilities remained low across the observed ranges. During Investigation 2, no significant associations between biomarkers and HAC were observed in either group. Progressive increases in plasma glutamine levels within the 31–60 and 8–30 days pre‐HAC window may serve as early markers of HAC risk in NO‐UCD, supporting the utility of longitudinal monitoring. These trends were not associated with LO‐UCD, suggesting the need for alternative surveillance strategies tailored to the onset phenotype.

## Summary

Longitudinal increases in plasma glutamine were associated with hyperammonemic crisis in neonatal‐onset urea cycle disorders.

## 1 Introduction

Urea cycle disorders (UCD) are inherited metabolic diseases caused by deficiencies in the hepatic urea cycle enzymes that convert toxic ammonia (NH_3_) to urea, leading to hyperammonemia [1]. The clinical phenotype of UCD varies depending on residual enzyme activity, ranging from neonatal‐onset (NO), which presents with acute symptoms such as recurrent vomiting, poor feeding, tachypnea, and altered consciousness within the first few days of life, to late‐onset (LO), in which neurological symptoms gradually emerge, often triggered by infections or prolonged fasting [1, 2].

Symptoms of UCD result from the neurotoxic effects of hyperammonemia, and plasma NH_3_ levels exceeding 600 μg/dL (352 μmol/L) have been associated with poor neurological outcomes [3]. Treatment strategies include acute interventions during catabolic stress and long‐term strategies such as dietary protein restriction, NH_3_‐scavenging agents, and supplementation with deficient amino acids [1, 4].

The primary goal of chronic management is to promote normal growth and development, while preventing acute hyperammonemic crises (HAC) [1, 5]. Prediction of HAC (NH_3_ > 150 μg/dL, 88.1 μmol/L) is essential for safe and effective care.

Current guidelines recommend maintaining plasma glutamine (Gln) concentrations below 1000 μmol/L, based on the observed correlation between Gln and NH_3_ levels [1, 6, 7]. However, in clinical practice, HAC can occur even in patients adhering to dietary and pharmacological therapies in accordance with these recommendations. Such episodes may be influenced by factors such as dietary compliance, growth rate, and relative changes in energy demand.

Fasting NH_3_ levels may be a better predictor of HAC than Gln [8, 9]; however, no consensus has been reached regarding the most reliable predictive biomarker. We aimed to identify the clinical markers that can predict the onset of hyperammonemia in patients with UCD undergoing chronic management. Recognizing the potential differences in disease severity and residual enzyme activity, we stratified patients by onset type—NO and LO [2, 10]. We investigated whether changes in plasma NH_3_ (ΔNH_3_) and Gln (ΔGln) concentrations, as measured during routine outpatient follow‐up, were associated with impending HAC and examined the time windows in which such associations might be observed.

## 2 Methods

### 2.1 Ethical Considerations

This study was conducted in accordance with the Declaration of Helsinki and approved by the Ethics Committee of Fujita Health University (approval no. HM25‐001). The study information was publicly disclosed on the university's official website, and potential participants were given an opportunity to opt out.

### 2.2 Study Design and Patients

This retrospective cohort study included patients with UCD treated at Fujita Health University between April 2014 and June 2024. All patients with confirmed biochemical and genetic diagnoses were classified into two groups according to the age at symptom onset: the NO group, in which symptoms manifested within the first 28 days of life, and the LO group, in which symptoms developed at 29 days of age or later. For patients who underwent liver transplantation, laboratory data obtained at least 8 days prior to transplantation were included in the analysis. All liver transplantations were planned living‐donor liver transplants (In Japan, most liver transplantations for UCD are performed as living‐donor procedures). Therefore, the pre‐transplant laboratory data were not related to acute HAC and were comparable to those obtained during routine outpatient follow‐up.

Treatment followed standard guidelines [1], including dietary protein restriction, supplementation with essential amino acids (EAA), and appropriate pharmacological interventions based on disease severity, avoidance of prolonged fasting, and management of catabolic stress. During chronic care, NO underwent monthly blood tests, LO every 2–3 months, and additional tests were performed during intercurrent illness.

### 2.3 Clinical and Laboratory Data

The following clinical variables were extracted from medical records: age, sex, UCD subtype, presence of neurological complications, mode of nutritional support, history of liver transplantation, age at initial symptom onset, intake of natural protein, use of EAA supplements, types and dosages of therapeutic agents, height, weight, and known triggers of HAC. Laboratory data included hemoglobin, total serum protein, albumin, total bilirubin, AST, ALT, BUN, plasma NH_3_ levels, and plasma amino acid concentrations (glutamate, Gln, glycine, alanine, citrulline [Cit], isoleucine, and arginine [Arg]). Laboratory data collected within 2 weeks of acute‐phase treatment, or within 2 months of the initial HAC were excluded from the analysis.

### 2.4 Biomarker Dynamics Analysis for HAC Prediction

HAC was defined as a plasma NH_3_ concentration > 150 μg/dL (88.1 μmol/L). ΔGln and ΔNH_3_ were calculated between sampling timepoints and analyzed in two investigations, stratified by NO/LO.

#### 2.4.1 Investigation 1

**FIGURE 1:**
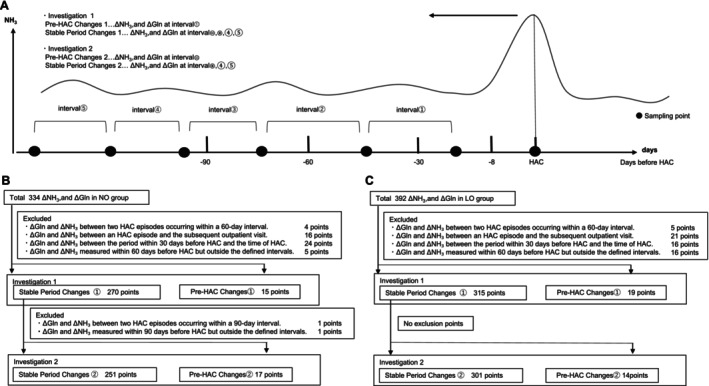
Study design and data selection for the analysis of changes in plasma glutamine (ΔGln) and changes in plasma ammonia (ΔNH_3_) in relation to hyperammonemic crisis (HAC), which was defined as plasma ammonia (NH_3_) concentration > 150 μg/dL (88.1 μmol/L). Caption: (A): Schematic representation of plasma NH_3_ fluctuations before HAC and the definition of time intervals used in two investigations. Black circles represent the blood sampling points. (B) A flowchart of data inclusion and exclusion in the neonatal‐onset (NO) group. (C) A flowchart of data inclusion and exclusion in the late‐onset (LO) group.

Comparison of ΔGln/ΔNH_3_ in Pre‐HAC Changes 1 and Stable Period Changes 1: Pre‐HAC Changes 1: ΔGln/ΔNH_3_ were measured between 31–60 days and 8–30 days before HAC (ΔGln/ΔNH_3_ interval (1) in Figure 1A).

Stable Period Changes 1: ΔGln/ΔNH_3_ measured between two regular visits temporally beyond the 31–60 days before HAC (ΔGln/ΔNH_3_ interval (2–5) in Figure 1A).

Exclusion Criteria for Investigation 1: ΔGln/ΔNH_3_ between two HAC episodes occurring within a 60‐day interval. ΔGln/ΔNH_3_ between an HAC episode and the subsequent outpatient visit. ΔGln/ΔNH_3_ was measured between the period within 30 days before HAC and the time of HAC. ΔGln/ΔNH_3_ measured within 60 days before HAC but outside the defined intervals (such as day −40 to day −5) (Figure 1B,C).

#### 2.4.2 Investigation 2

Pre‐HAC Changes 2: ΔGln/ΔNH_3_ between 61–90 and 31–60 days before HAC (interval (2) in Figure 1A).

Stable Period Changes 2: ΔGln/ΔNH_3_ between two routine visits occurring outside the 61–90‐day pre‐HAC window (intervals (3–5) in Figure 1A).

Exclusion criteria: All exclusions from Investigation 1 apply, and additionally: Two HAC episodes occurring within a 90‐day interval, and ΔGln/ΔNH_3_ measured within 90 days before HAC but outside the defined windows (Figure 1B,C).

### 2.5 Statistical Analysis

As multiple observations per patient were available, we fitted generalized linear mixed‐effects models (generalized linear mixed models [GLMM]; binomial family with logit link) to evaluate the association between biomarker changes and the occurrence of hyperammonemia, allowing for effect modification by group (NO/LO). Two prespecified models were estimated separately: one with ΔGln and one with ΔNH_3_ as the biomarker of interest. In each model, fixed effects included the biomarker (ΔGln or ΔNH_3_), group, and their interaction (biomarker × group). Random effects comprised patient‐specific intercepts and slopes for within‐patient biomarker changes, thereby accounting for the between‐patient heterogeneity in baseline risk and biomarker responsiveness. From each fitted model, we obtained marginal estimated probabilities of hyperammonemia for prespecified values of ΔGln or ΔNH_3_ within each group, with 95% confidence intervals derived from the model variance–covariance matrix. We assessed potential overfitting of the mixed‐effects logistic regression model using internal validation by cluster bootstrap at the participant (ID) level with 150 resamples. For each bootstrap resample, the model was refitted and the calibration slope on the logit scale was estimated both in the bootstrap sample (apparent performance) and in the original dataset using the bootstrap‐fitted model; optimism was defined as the mean difference between these slopes across resamples. An optimism‐corrected calibration slope was obtained by subtracting the estimated optimism from the apparent calibration slope, and we considered optimism ≤ 0.2 as indicating no evidence of overfitting. Given the small sample size, the baseline characteristics were presented as individual case narratives rather than as aggregated summaries. All tests were two‐sided, with a significance level of 0.05. All analyses were conducted using R version 4.4.1 (R Foundation for Statistical Computing, Vienna, Austria).

## 3 Results

### 3.1 Patient Characteristics

Patient characteristics are summarized in Table 1, with detailed data provided in Table S1A for the NO group and Table S1B for the LO group. Among the 18 patients, NO (*n* = 9) included four with carbamoyl‐phosphate synthetase 1 deficiency (CPS1D; OMIM #237300), four with ornithine transcarbamylase deficiency (OTCD; OMIM #311250), and one with argininosuccinate synthetase deficiency (ASSD; OMIM #215700); LO (*n* = 9) included eight with OTCD and one with ASSD. The male‐to‐female ratio in the NO group was 5:4. In the LO group, all patients were female. The median natural protein intake was 0.47 and 0.79 g/kg/day in NO and LO, respectively. Oral supplementation with EAA was 0.22 g/kg/day in NO; in LO, EAA supplementation was generally 0 g/kg/day, with only three patients receiving minimal doses. The feeding route in patients in the NO group was one oral‐only, four oral with supplemental tube, and four tube‐only; in patients in the LO group, eight were oral‐only, and one was tube‐only. Neurological complications occurred in seven patients in the NO group (four with cerebral palsy) and in one patient with LO (cerebral palsy). Liver transplantation was performed in five patients in the NO group and in none in the LO group. The median daily doses of NO were sodium benzoate 152 mg/kg/day, sodium phenylbutyrate 173 mg/kg/day, L‐Arg 127 mg/kg/day, and L‐Cit 148 mg/kg/day; in LO, sodium benzoate 0 mg/kg/day, sodium phenylbutyrate 207 mg/kg/day, L‐Arg 105 mg/kg/day, and L‐Cit 109 mg/kg/day.

**TABLE 1:** Clinical characteristics of patients with neonatal‐onset (NO) and late‐onset (LO) urea cycle disorders.

	Neonatal‐onset	Late‐onset
Diagnosis (*n*)	OTCD: 4, CPS‐1D: 4, ASSD: 1	OTCD: 8, ASSD: 1
Sex (M:F)	5:4	0:9
Age at sampling (years) Median (IQR)	14 (2–20)	18 (8–22)
Natural protein intake (g/kg) Median (IQR)	0.47 (0.32–0.71)	0.79 (0.63–1.04)
EAA (g/kg) Median (IQR)	0.22 (0.13–0.32)	0 (0–0.12)
Sodium Benzoate (mg/kg) Median (IQR)	152 (143–178)	0 (0–112)
Sodium phenylbutyrate (mg/kg) Median (IQR)	173 (109–229)	207 (145–242)
L‐arginine (mg/kg) Median (IQR)	127 (0–160)	105 (82–145)
L‐citrulline (mg/kg) Median (IQR)	148 (105–189)	109 (73–150)
Liver transplantation (*n*)	5	0
Nutrition method (*n*)	Gastrostomy nutrition: 4, Oral intake: 5 (4 with NG tube)	Gastrostomy nutrition: 1, Oral intake: 8
Neurological complications (*n*)	Cerebral palsy: 4, Intellectual disability: 3, Epilepsy: 1	Cerebral palsy: 1, Epilepsy: 1

Abbreviations: EAA, essential amino acids; IQR, interquartile range; NG tube, nasogastric tube.

### 3.2 Frequency of HAC Episodes and Presumed Causes

In the NO group, 24 HAC episodes were recorded over 29 patient‐years (events from eight patients; one experienced none). In the LO group, 39 episodes occurred over 59 patient‐years (events in eight patients; one had none). Peak NH_3_ values and annual HAC frequency are summarized in Tables S2A and S2B. The reasons for HAC were excess protein (*n* = 9), catabolic stress (*n* = 13), and unknown (*n* = 2) in the NO group, and excess protein (*n* = 18), catabolic stress (*n* = 9), poor adherence (*n* = 2), and unknown (*n* = 10) in the LO group.

### 3.3 Results of ΔGln/ΔNH_3_ as Predictors of HAC Across 31–60 → 8–30 (Investigation 1) and 61–90 → 31–60 Days (Investigation 2)

The number of pre‐HAC observations contributing to each ΔGln and ΔNH_3_ range in Investigation 1, stratified by onset type, is presented in Table S3.

#### 3.3.1 Results of Investigation 1: ΔGln

GLMM showed ΔGln was associated with increased HAC risk in NO (*p* < 0.001) but not LO (*p* = 0.470); the ΔGln × onset interaction was significant (*p* < 0.01), indicating that the association between ΔGln and HAC varied depending on the onset type. A total of 619 records were included in the analysis, and the estimated optimism was 0.134.

**FIGURE 2:**
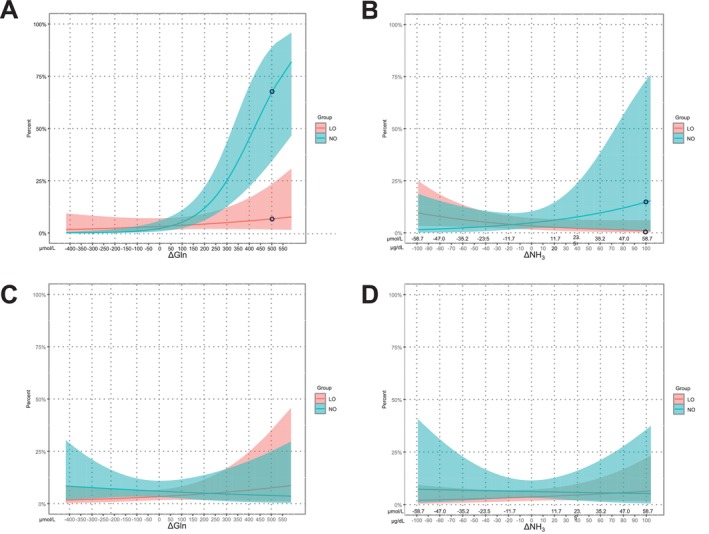
HAC probability based on ΔGln and ΔNH_3_ Caption: The blue line indicates the median, and the shaded blue band indicates the 95% confidence interval (NO). The red line indicates the median, and the shaded red band indicates the 95% confidence interval (LO). (A) Investigation 1, probability of HAC to Gln in the NO (blue) and LO (red) groups (between 31–60 and 8–30 days prior to HAC). ∘ Indicates the mean probability in NO and LO at ΔGln +500 μmol/L. (B) Investigation 1, probability of HAC to ΔNH_3_ (31–60 and 8–30 days prior to HAC). ∘ Indicates the mean probability in NO and LO at ΔNH_3_ +100 μg/dL (58.7 μmol/L). (C) Investigation 2, probability of HAC to ΔGln (61–90 and 31–60 days prior to HAC). (D) Investigation 2, probability of HAC to ΔNH_3_ (61–90 and 31–60 days prior to HAC).

In the NO group (blue band), the probability of HAC rose sharply with ΔGln, reaching 67.1% at ΔGln = 500 μmol/L (Figure 2A and Table S4A). In LO (red band), the probability of HAC remained low across −400 μmol/L to +550 μmol/L, at 6.70% at ΔGln = 500 μmol/L.

#### 3.3.2 Results of Investigation 1: ΔNH_3_

GLMM showed ΔNH_3_ was not associated with increased HAC risk in NO (*p* = 0.361), and the ΔNH_3_ × onset interaction was not significant (*p* = 0.117), indicating that the effect of ΔNH_3_ did not significantly differ between NO and LO. A total of 619 records were included in the analysis, and the estimated optimism was 0.400. HAC probability in the NO group increased with ΔNH_3_, reaching 14.7% at ΔNH_3_ = 100 μg/dL (58.7 μmol/L) (Figure 2B and Table S4B). In LO, the HAC probability remained low, decreasing from 9.60% at ΔNH_3_ = −100 μg/dL (−58.7 μmol/L) to 1.09% at ΔNH_3_ = 100 μg/dL (58.7 μmol/L).

#### 3.3.3 Results of Investigation 2: ΔGln

No significant ΔGln × onset interaction (*p* = 0.326) was observed. HAC probability was similar between groups and largely unchanged across ΔGln, indicating limited association (Figure 2C). A total of 583 records were included in the analysis, and the estimated optimism was 0.327.

#### 3.3.4 Results of Investigation 2: ΔNH_3_

No significant ΔNH_3_ × onset interaction (*p* = 0.559) was observed. Neither group showed meaningful change in HAC probability with ΔNH_3_, again indicating limited association (Figure 2D). A total of 583 records were included in the analysis, and the estimated optimism was 0.433.

## 4 Discussion

This study examined whether temporal ΔGln and ΔNH_3_ concentrations were associated with HAC in patients with UCD, comparisons of NO and LO phenotypes. A distinct temporal window of 31–60 and 8–30 days before HAC onset was identified, during which increases in plasma Gln were associated with HAC in NO. These findings suggest that the magnitude of the increase in Gln may provide a greater prognostic value than absolute concentrations alone in the NO group. In contrast, in the LO group, changes in Gln and NH_3_ were not significantly associated with HAC. These differences may reflect variations in residual enzymatic activity and metabolic reserves [2, 10].

The NO group included more severe cases, had a lower total protein intake, and more frequently received NH_3_ scavenger therapy and amino acid supplementation. While NH_3_ levels were strictly controlled in the NO group, 24 HAC occurred over 29 patient‐years in nine individuals. In contrast, the LO group experienced 39 episodes over 59 patient‐years in nine individuals. Complete prevention of hyperammonemia was not achieved in the patients with NO‐UCD [11]. However, the 10‐year individual laboratory data from our institution (Tables S5A and S5B) suggest that relatively lower plasma Gln levels, compared with the current guideline targets, may have contributed to the partial control of HAC occurrence in this more severely affected population. Patients with NO‐UCD, who typically require strict metabolic control, may be more vulnerable to relatively modest elevations in biochemical markers. In contrast, greater metabolic flexibility in LO‐UCD may obscure early changes, and symptoms may be mitigated or managed through informal dietary adjustments, resulting in the underrecognition of early HAC episodes [12].

Previous studies have predominantly focused on the baseline values of Gln and NH_3_, with plasma Gln concentrations exceeding 900–1000 μmol/L considered associated with an increased risk of HAC [1, 6, 7]. However, few studies have investigated the predictive value of these biomarkers for fluctuations. Only one study has reported that even a 100–200 μmol/L increase in Gln did not significantly elevate the risk of hyperammonemia within 3 months, and that fasting baseline NH_3_ was a more reliable predictor [8]. The present findings support the utility of close, longitudinal monitoring of plasma Gln levels in patients in the NO group to facilitate early identification of at‐risk periods and to guide timely interventions. For example, when a marked increase in plasma Gln is detected during routine follow‐up, clinicians may consider advancing the next outpatient visit and modifying dietary therapy (such as short‐term dietary protein restriction), as well as adjusting the dose of ammonia scavenger therapy. However, for patients in the LO group, these markers appear less informative, and alternative approaches that incorporate clinical symptoms, broader metabolic profiles, or novel biomarkers may be needed to improve risk stratification.

This study had some limitations. This retrospective design may introduce bias owing to the non‐standardized timing of blood sampling and the possibility that treatment adjustments were made in response to interim laboratory results, potentially confounding the association between biomarker changes and HAC. Additionally, the observed optimism suggests that model performance may have been overestimated, likely reflecting potential overfitting due to the limited sample size, the predominance of proximal UCD subtypes, and the clustering of HAC events within three or fewer participants in both the NO and LO groups. Accordingly, the observed associations, or lack thereof, may reflect the biochemical profiles of these individual patients rather than those of the NO or LO groups. The younger age of patients in the NO group, partly due to early liver transplantation, and differences in clinical follow‐up or adherence to therapy, could also have influenced the outcomes. To overcome these limitations, larger multicenter studies will be needed to obtain more representative longitudinal datasets in patients with UCD.

While this study focused on routinely collected laboratory parameters, the ability to monitor plasma NH_3_ and Gln levels more frequently may enhance clinical risk assessment, particularly in patients with NO‐UCD. Recent technological developments have led to the exploration of home‐based measurement devices for plasma NH_3_ and, to a lesser extent, plasma Gln [13, 14]. Such methods can facilitate the earlier detection of metabolic instability during the pre‐crisis phase; however, they remain to be widely implemented. Further prospective studies are required to assess the clinical applicability, accuracy, and feasibility of these methods in the outpatient setting.

## 5 Conclusion

In NO‐UCD, rising plasma Gln levels between 31–60 and 8–30 days prior to HAC may serve as early indicators. However, these markers were not significantly associated with LO cases within this cohort, given the limitations of the present sample size and less frequent sampling, suggesting that onset‐specific monitoring strategies may be needed.

## Author Contributions

**Yasuaki Yasuda:** methodology, data curation, and writing – original draft. **Yoko Nakajima:** conceptualization, supervision. **Yuta Sudo:** data curation. **Takuma Ishihara:** formal and statistical analyses. **Tetsuya Ito:** writing – review and editing; critical revision of the manuscript.

## Funding

The authors have nothing to report.

## Ethics Statement

This study was conducted in accordance with the principles of the Declaration of Helsinki and approved by the Ethics Committee of Fujita Health University (Approval No. HM25‐001).

## Consent

The study information was publicly disclosed on the university's official website, and potential participants were given an opportunity to opt out.

## Conflicts of Interest

The authors declare no conflicts of interest.

## Supporting information


**Table S1A:** Characteristics of the patients in the neonatal‐onset (NO) group.
**Table S1B:** Characteristics of patients in the late‐onset (LO) group.
**Table S2A:** Annual frequency of hyperammonemic crisis (HAC) and peak plasma ammonia (NH_3_) levels in the neonatal‐onset (NO) group.
**Table S2B:** Annual frequency of HAC and peak plasma NH_3_ levels in the late‐onset (LO) group.
**Table S3:** Number of pre‐HAC observations contributing to each ΔGln and ΔNH_3_ range by onset type (neonatal‐onset (NO) vs. late‐onset (LO)) in Investigation 1.
**Table S4A:** Predicted probability of HAC by change in plasma glutamine (ΔGln).
**Table S4B:** Predicted probability of HAC by change in plasma ammonia (ΔNH_3_).
**Table S5A:** Laboratory parameters during the stable period in the NO group.
**Table S5B:** Laboratory parameters during the stable period in the LO group.

## Data Availability

The data that support the findings of this study are available from the corresponding author upon reasonable request.

## References

[1] J. Häberle , N. Boddaert , A. Burlina , et al., “Suggested Guidelines for the Diagnosis and Management of Urea Cycle Disorders: First Revision,” Journal of Inherited Metabolic Disease 42, no. 6 (2019): 1192–1230, 10.1002/jimd.12100. 30982989

[2] R. Posset , S. F. Garbade , N. Boy , et al., “Transatlantic Combined and Comparative Data Analysis of 1095 Patients With Urea Cycle Disorders—A Successful Strategy for Clinical Research of Rare Diseases,” Journal of Inherited Metabolic Disease 42, no. 1 (2019): 93–106, 10.1002/jimd.12031. 30740724 PMC7329920

[3] J. Kido , S. Matsumoto , J. Häberle , et al., “Long‐Term Outcome of Urea Cycle Disorders: Report From a Nationwide Study in Japan,” Journal of Inherited Metabolic Disease 44, no. 3 (2021): 673–684, 10.1002/jimd.12384. 33840128

[4] R. Posset , S. F. Garbade , F. Gleich , et al., “Long‐Term Effects of Medical Management on Growth and Weight in Individuals With Urea Cycle Disorders,” Scientific Reports 10, no. 1 (2020): 11948, 10.1038/s41598-020-67496-3. 32686765 PMC7371674

[5] J. Häberle , “Primary Hyperammonaemia: Current Diagnostic and Therapeutic Strategies,” Journal of Mother and Child 24, no. 2 (2020): 32–38, 10.34763/jmotherandchild.20202402si.2015.000006. 33179600 PMC8518097

[6] N. E. Maestri , K. D. McGowan , and S. W. Brusilow , “Plasma Glutamine Concentration: A Guide in the Management of Urea Cycle Disorders,” Journal of Pediatrics 121, no. 2 (1992): 259–261, 10.1016/s0022-3476(05)81200-4. 1640294

[7] C. J. Wilson , P. J. Lee , and J. V. Leonard , “Plasma Glutamine and Ammonia Concentrations in Ornithine Carbamoyltransferase Deficiency and Citrullinaemia,” Journal of Inherited Metabolic Disease 24, no. 6 (2001): 691–695, 10.1023/A:1012995701589. 11804205

[8] B. Lee , G. A. Diaz , W. Rhead , et al., “Blood Ammonia and Glutamine as Predictors of Hyperammonemic Crises in Patients With Urea Cycle Disorder,” Genetics in Medicine 17, no. 7 (2015): 561–568, 10.1038/gim.2014.148. 25503497 PMC4465427

[9] B. Lee , G. A. Diaz , W. Rhead , et al., “Glutamine and Hyperammonemic Crises in Patients With Urea Cycle Disorders,” Molecular Genetics and Metabolism 117, no. 1 (2016): 27–32, 10.1016/j.ymgme.2015.11.005. 26586473 PMC4915945

[10] S. Scharre , R. Posset , S. F. Garbade , et al., “Predicting the Disease Severity in Male Individuals With Ornithine Transcarbamylase Deficiency,” Annals of Clinical Translational Neurology 9, no. 11 (2022): 1715–1726, 10.1002/acn3.51668. 36217298 PMC9639638

[11] C. Unsinn , A. Das , V. Valayannopoulos , et al., “Clinical Course of 63 Patients With Neonatal Onset Urea Cycle Disorders in the Years 2001–2013,” Orphanet Journal of Rare Diseases 11, no. 1 (2016): 116, 10.1186/s13023-016-0493-0. 27538463 PMC4991093

[12] C. M. Rüegger , M. Lindner , D. Ballhausen , et al., “Cross‐Sectional Observational Study of 208 Patients With Non‐Classical Urea Cycle Disorders,” Journal of Inherited Metabolic Disease 37, no. 1 (2014): 21–30, 10.1007/s10545-013-9624-0. 23780642 PMC3889631

[13] J. M. Fletcher and D. W. Johnson , “Quantification of Glutamine in Dried Blood Spots and Plasma by Tandem Mass Spectrometry for the Biochemical Diagnosis and Monitoring of Ornithine Transcarbamylase Deficiency,” Clinical Chemistry 49, no. 4 (2003): 681, 10.1373/49.4.681. 12651832

[14] J. P. Talley , T. J. Free , T. P. Green , D. M. Chipman , and B. C. Bundy , “Eliminating Assay Background of a Low‐Cost, Colorimetric Glutamine Biosensor by Engineering an Alternative Formulation of Cell‐Free Protein Synthesis,” Chem 13, no. 6 (2025): 206, 10.3390/chemosensors13060206.

