# Supplementary material for: Longitudinal Changes in Glutamine and Ammonia in Relation to Hyperammonemic Crisis in Urea Cycle Disorders
Source: JIMD Rep. 2026 Jun 18;67(4):e70105. doi: 10.1002/jmd2.70105 (PMC13276496; doi:10.1002/jmd2.70105)
Supplement: Supplementary file 1 — Table S1A: Characteristics of the patients in the neonatal‐onset (NO) group. Table S1B: Characteristics of patients in the late‐onset (LO) group. Table S2A: Annual frequency of hyperammonemic crisis (HAC) and peak plasma ammonia (NH3) levels in the neonatal‐onset (NO) group. Table S2B: Annual frequency of HAC and peak plasma NH3 levels in the late‐onset (LO) group. Table S3: Number of pre‐HAC observations contributing to each ΔGln and ΔNH3 range by onset type (neonatal‐onset (NO) vs. late‐onset (LO)) in Investigation 1. Table S4A: Predicted probability of HAC by change in plasma glutamine (ΔGln). Table S4B: Predicted probability of HAC by change in plasma ammonia (ΔNH3). Table S5A: Laboratory parameters during the stable period in the NO group. Table S5B: Laboratory parameters during the stable period in the LO group. [file JMD2-67-e70105-s001.docx]

Supplementary Table S1-A: Characteristics of the patients in the neonatal-onset (NO) group

| No | Diagnosis | Sex | Age at  blood sampling | Natural protein (g/kg)  median  (IQR) | EAA (g/kg)  median  (IQR) | NaBZ (mg/kg)  median  (IQR) | NaPB (mg/kg)  median  (IQR) | L-Arg (mg/kg)  median  (IQR) | L-Cit (mg/kg)  median  (IQR) | Liver  transplant | Neurological  complications | Nutrition method |
| --- | --- | --- | --- | --- | --- | --- | --- | --- | --- | --- | --- | --- |
| 1 | CPS1D | F | 13y-22y | 0.19 | 0.066 | 155 | 0 | 0 | 124 | - | cerebral palsy | Gastrostomy nutrition |
|  |  |  |  | (0.17-0.23) | (0.064-0.11) | (152-156) | (0-156) | (0-40.0) | (118-130) |  |  |  |
| 2 | CPS1D | F | 2m-7y | 0.6 | 0.33 | 178 | 188 | 158 | 181 | - | mental retardation | Oral intake + Gastrostomy nutrition |
|  |  |  |  | (0.49-0.69) | (0.23-0.38) | (161-189) | (175-226) | (123-185) | (108-190) |  |  |  |
| 3 | OTCD | M | 2m-1y | 1.04 | 0.36 | - | 139 | - | 0 | + | mental retardation | Oral intake + |
|  |  |  |  | (0.98-1.09) | (0.30-0.38) |  | (134-150) |  | (0-0) |  |  | NG tube |
| 4 | OTCD | M | 2m-11m | 0.95 | 0.38 | 157 | 188 | 163 | 200 | + | cerebral palsy | Gastrostomy nutrition |
|  |  |  |  | (0.86-0.97) | (0.32-0.42) | (0-186) | (186-225) | (154-164) | (197-205) |  |  |  |
| 5 | OTCD | M | 2m-10m | 0.53 | 0.25 | - | 118 | - | 171 | + | cerebral palsy | Gastrostomy nutrition |
|  |  |  |  | (0.31-0.67) | (0.24-0.26) |  | (114-123) |  | (169-181) |  |  |  |
| 6 | ASSD | M | 14y-17y | 0.77 | 0.27 | 195 | 276 | 279 | - | - | cerebral palsy | Gastrostomy nutrition |
|  |  |  |  | (0.76-0.81) | (0.27-0.28) | (192-208) | (272-288) | (274-297) |  |  | epilepsy |  |
| 7 | OTCD | M | 2m-1y | 0.73 | 0.31 | 184 | 232 | 139 | 129 | + | - | Oral intake + |
|  |  |  |  | (0.57-0.86) | (0.28-0.35) | (145-192) | (213-247) | (133-148) | (102-146) |  |  | NG tube |
| 8 | CPS1D | F | 19y-28y | 0.41 | 0.13 | 144 | 116 | 133 | 190 | - | mental retardation | Oral intake |
|  |  |  |  | (0.37-0.45) | (0.13-0.14) | (143-148) | (87-225) | (125-146) | (184-195) |  |  |  |
| 9 | CPS1D | F | ２m-1y | 0.62 | 0.32 | 143 | 229 | 92 | 88 | + | - | Oral intake + |
|  |  |  |  | (0.58-0.81) | (0.30-0.35) | (128-169) | (211-246) | (76-97) | (84-94) |  |  | NG tube |
| Median |  | M: 5 |  | 0.47 | 0.22 | 152 | 173 | 127 | 148 |  |  |  |
|  |  | F: 4 |  | (0.32-0.71) | (0.13-0.32) | (143-178) | (109-229) | (0-160) | (105-189) |  |  |  |

IQR, interquartile range; EAA, essential amino acids; NaBZ, sodium benzoate; NaPB, sodium phenylbutyrate; L-Arg, L-arginine; L-Cit, L-citrulline; NG tube, nasogastric tube.

Supplementary Table S1-B: Characteristics of patients in the late-onset (LO) group

| No | Diagnosis | Sex | Age at  blood sampling | Natural protein (g/kg)  median  (IQR) | EAA (g/kg)  median  (IQR) | NaBZ (mg/kg)  median  (IQR) | NaPB (mg/kg)  median  (IQR) | L-Arg (mg/kg)  median  (IQR) | L-Cit (mg/kg)  median  (IQR) | Liver  transplant | Neurological  complications | Nutrition method |
| --- | --- | --- | --- | --- | --- | --- | --- | --- | --- | --- | --- | --- |
| 1 | OTCD | F | 21y-26y | 0.81 | - | - | 187 | 83 | 108 | - | - | Oral intake |
|  |  |  |  | (0.74-0.98) |  |  | (177-220) | (81.9-84.1) | (107-109) |  |  |  |
| 2 | OTCD | F | 17y-25y | 1.04 | - | - | 207 | 95 | 89 | - | - | Oral intake |
|  |  |  |  | (1.03-1.15) |  |  | (201-229) | (0-102) | (88-159) |  |  |  |
| e | OTCD | F | 21y-24y | 0.69 | - | 107 | 225 | 146 | - | - | - | Oral intake |
|  |  |  |  | (0.61-0.77) |  | (106-109) | (205-228) | (144-161) |  |  |  |  |
| 4 | OTCD | F | 3y-11y | 0.78 | 0.09 | 177 | 215 | 136 | 142 | - | - | Oral intake |
|  |  |  |  | (0.63-0.99) | (0.08-0.13) | (0-193) | (173-237) | (0-156) | (71-167) |  |  |  |
| 5 | OTCD | F | 19y-27y | 1.59 | - | - | 85 | 95 | 103 | - | - | Oral intake |
|  |  |  |  | (1.30-1.67) |  |  | (59-97) | (75-96) | (78-125) |  |  |  |
| 6 | OTCD | F | 10y-19y | 0.6 | 0.21 | 0 | 191 | 108 | 147 | - | cerebral palsy | Gastrostomy nutrition |
|  |  |  |  | (0.57-0.64) | (0.17-0.22) | (0-176) | (148-242) | (52-121) | (139-161) |  | epilepsy |  |
| 7 | ASSD | F | 5y-11y | 1.6 | - | - | 70 | 186 | - | - | - | Oral intake |
|  |  |  |  | (1.56-1.73) |  |  | (0-136) | (149-197) |  |  |  |  |
| 8 | OTCD | F | 26y-30y | 0.75 | - | 73 | 172 | 93 | 99 | - | - | Oral intake |
|  |  |  |  | (0.65-0.80) |  | (48-74) | (162-178) | (85-96) | (97-104) |  |  |  |
| 9 | OTCD | F | 19y-28y | 0.83 | 0.08  (0.08 | 49 | 246 | 92 | 143 | - | - | Oral intake |
|  |  |  |  | (0.58-0.83) | -0.08) | (0-143) | (224-291) | (0-110) | (143-218) |  |  |  |
| median |  | M 0 |  | 0.79 | 0 | 0 | 207 | 105 | 109 |  |  |  |
| (IQR) |  | F 9 |  | (0.63-1.04) | (0-0.12) | (0-112) | (145-242) | (82-145) | (73-150) |  |  |  |

IQR, interquartile range; EAA, essential amino acids; NaBZ, sodium benzoate; NaPB, sodium phenylbutyrate; L-Arg, L-arginine; L-Cit, L-citrulline; NG tube, nasogastric tube.

Supplementary Table S2-A. Annual Frequency of Hyperammonemic Crisis (HAC) and Peak Plasma Ammonia (NH_3_) Levels in the Neonatal-Onset (NO) group

| NO group | | | | | |
| --- | --- | --- | --- | --- | --- |
| No | age | HAC  frequency | Peak plasma NH_3_  at HAC (μg/dL) | | |
| 1 | 13 | 1 | 256 |  |  |
| 1 | 14–17 | 0 |  |  |  |
| 1 | 18 | 1 | 280 |  |  |
| 1 | 19–21 | 0 |  |  |  |
| 2 | 0 | 3 | 189 | 199 | 245 |
| 2 | 1 | 3 | 317 | 227 | 496 |
| 2 | 6 | 1 | 306 |  |  |
| 3 | 0 | 1 | 161 |  |  |
| 4 | 0 | 0 |  |  |  |
| 5 | 0 | 2 | 200 | 439 |  |
| 6 | 14 | 1 | 165 |  |  |
| 6 | 15–16 | 0 |  |  |  |
| 7 | 0 | 1 | 280 |  |  |
| 8 | 19 | 1 | 174 |  |  |
| 8 | 20 | 1 | 279 |  |  |
| 8 | 21 | 1 | 372 |  |  |
| 8 | 22 | 0 |  |  |  |
| 8 | 23 | 2 | 193 | 180 |  |
| 8 | 24 | 2 | 189 | 320 |  |
| 8 | 25 | 2 | 288 | 206 |  |
| 8 | 26–27 | 0 |  |  |  |
| 9 | 0 | 1 | 230 |  |  |

Annual frequency of HAC (NH_3_>150 μg/dL, 88.1 μmol/L) episodes and corresponding peak plasma NH₃ concentrations (μg/dL) for each year of life in patients in the NO group. Blank entries indicate the years without HAC episodes.

Supplementary Table S2-B. Annual Frequency of HAC and Peak Plasma NH_3_ Levels in the Late-Onset (LO) group

| LO group | | | | | | | |
| --- | --- | --- | --- | --- | --- | --- | --- |
| No | age | HAC  frequency | Peak plasma NH_3_ at HAC (μg/dL) | | | | |
| 1 | 21 | 1 | 162 |  |  |  |  |
| 1 | 22 | 1 | 204 |  |  |  |  |
| 1 | 23 | 2 | 163 | 169 |  |  |  |
| 1 | 24 | 0 |  |  |  |  |  |
| 1 | 25 | 2 | 162 | 222 |  |  |  |
| 1 | 26 | 0 |  |  |  |  |  |
| 2 | 17 | 1 | 299 |  |  |  |  |
| 2 | 18–21 | 0 |  |  |  |  |  |
| 2 | 22 | 1 | 165 |  |  |  |  |
| 2 | 23 | 0 |  |  |  |  |  |
| 3 | 21 | 1 | 192 |  |  |  |  |
| 3 | 22 | 1 | 219 |  |  |  |  |
| 3 | 23 | 0 |  |  |  |  |  |
| 4 | 3 | 5 | 158 | 198 | 157 | 245 | 180 |
| 4 | 4 | 5 | 203 | 160 | 173 | 191 | 349 |
| 4 | 5 | 3 | 229 | 196 | 417 |  |  |
| 4 | 6 | 3 | 241 | 376 | 234 |  |  |
| 4 | 7 | 0 |  |  |  |  |  |
| 4 | 8 | 4 | 566 | 466 | 453 | 718 |  |
| 4 | 9 | 1 | 174 |  |  |  |  |
| 4 | 10 | 0 |  |  |  |  |  |
| 5 | 19 | 1 | 208 |  |  |  |  |
| 5 | 20–25 | 0 |  |  |  |  |  |
| 6 | 10–17 | 0 |  |  |  |  |  |
| 6 | 18 | 1 | 198 |  |  |  |  |
| 7 | 5–11 | 0 |  |  |  |  |  |
| 8 | 25 | 1 | 256 |  |  |  |  |
| 8 | 26–29 | 0 |  |  |  |  |  |
| 9 | 18 | 1 | 192 |  |  |  |  |
| 9 | 19 | 2 | 232 | 318 |  |  |  |
| 9 | 20 | 0 |  |  |  |  |  |
| 9 | 21 | 1 | 187 |  |  |  |  |
| 9 | 22 | 0 |  |  |  |  |  |
| 9 | 23 | 1 | 169 |  |  |  |  |
| 9 | 24–25 | 0 |  |  |  |  |  |

Annual frequency of HAC (NH_3_>150 μg/dL, 88.1 μmol/L) episodes and corresponding peak plasma NH₃ concentrations (μg/dL) for each year of life in patients with the LO group. Blank entries indicate the years without HAC episodes.

Supplementary Table S3. Number of pre-HAC observations contributing to each ΔGln and ΔNH₃ range by onset type (Neonatal-Onset (NO) vs Late-Onset (LO)) in Investigation 1.

| ΔGln range (μmol/L) | NO  (samples) | LO  (samples) | ΔNH₃ range (μg/dL) | NO  (samples) | LO  (samples) |
| --- | --- | --- | --- | --- | --- |
| -800 to -600 | 1 | 0 | -120 to -80 | 1 | 10 |
| -600 to -400 | 2 | 3 | -80 to -40 | 11 | 38 |
| -400 to -200 | 10 | 43 | -40 to 0 | 140 | 128 |
| -200 to 0 | 116 | 119 | 0 to 40 | 120 | 105 |
| 0 to 200 | 135 | 130 | 40 to 80 | 12 | 43 |
| 200 to 400 | 13 | 37 | 80 to 120 | 1 | 9 |
| 400 to 800 | 8 | 2 | 120 to 149 | 0 | 1 |

Supplementary Table S4-A. Predicted Probability of HAC by Change in Plasma Glutamine (ΔGln)

|  | NO group |  |  | LO group |  |
| --- | --- | --- | --- | --- | --- |
| ΔGln (μmol/L) | Prediction (%) | 95% CI | ΔGln (μmol/L) | Prediction (%) | 95% CI |
| -400 | 0.059 | 0.0057 – 0.61 | -400 | 1.69 | 0.290 – 9.19 |
| -300 | 0.15 | 0.020 – 1.06 | -300 | 1.97 | 0.45 – 8.27 |
| -200 | 0.36 | 0.070 – 1.84 | -200 | 2.30 | 0.68 – 7.45 |
| -100 | 0.89 | 0.24 – 3.24 | -100 | 2.69 | 0.99 – 7.11 |
| 0 | 2.16 | 0.78 – 5.84 | 0 | 2.16 | 1.36 – 7.06 |
| 100 | 5.18 | 2.37 – 11.0 | 100 | 3.66 | 1.71 – 7.65 |
| 200 | 11.9 | 6.09 – 22.0 | 200 | 4.27 | 1.91 – 9.25 |
| 300 | 25.0 | 12.7 – 43.5 | 300 | 4.96 | 1.93 – 12.2 |
| 400 | 45.2 | 22.2 – 70.5 | 400 | 5.77 | 1.82 – 16.8 |
| 500 | 67.1 | 34.4 – 88.8 | 500 | 6.70 | 1.67 – 23.4 |

Estimated probability of HAC (%) and 95% confidence intervals (CI) are presented for each ΔGln value (between 31–60 and 8–30 days before HAC onset) in the NO and LO groups.
 The predicted probabilities were derived from the GLMM with group-specific effects.

Supplementary Table S4-B. Predicted Probability of HAC by Change in Plasma Ammonia (ΔNH₃)

|  | NO group |  |  | LO group |  |
| --- | --- | --- | --- | --- | --- |
| ΔNH_3_ (μg/dL) | Prediction (%) | 95% CI | ΔNH_3_ (μg/dL) | Prediction (%) | 95% CI |
| -100 | 1.45 | 0.093 – 18.9 | -100 | 9.60 | 3.22 – 25.3 |
| -80 | 1.85 | 0.20 – 15.3 | -80 | 7.81 | 3.09 – 18.4 |
| -60 | 2.35 | 0.40 – 12.5 | -60 | 6.33 | 2.86 – 13.4 |
| -40 | 2.99 | 0.80 – 10.5 | -40 | 5.11 | 2.50 – 10.2 |
| -20 | 3.80 | 1.47 – 9.47 | -20 | 4.12 | 2.02 – 8.22 |
| 0 | 4.80 | 2.17 – 10.28 | 0 | 3.31 | 1.50 – 7.13 |
| 20 | 6.06 | 2.38 – 14.6 | 20 | 2.66 | 1.05 – 6.55 |
| 40 | 7.63 | 2.13 – 23.9 | 40 | 2.13 | 0.71 – 6.25 |
| 60 | 9.55 | 1.75 – 38.5 | 60 | 1.71 | 0.46 – 6.09 |
| 80 | 11.9 | 1.39 – 56.4 | 80 | 1.36 | 0.30 – 6.03 |
| 100 | 14.7 | 1.08 – 73.2 | 100 | 1.09 | 0.19 – 6.02 |

Estimated probability of HAC (%) and 95% CI for each ΔNH₃ value (NH_3_ between 31–60 and 8–30 days before HAC onset) in the NO and LO groups.

Supplementary Table S5-A. Laboratory Parameters During the Stable Period in the NO Group

| NO group | | | | | | | |
| --- | --- | --- | --- | --- | --- | --- | --- |
| No | TP (g/dL) | Alb (g/dL) | AST (IU/L) | ALT (IU/L) | NH₃  (μg/dL) | Glu (μmol/L) | Gln (μmol/L) |
|  | median | median | median | median | median | median | median |
|  | (IQR) | (IQR) | (IQR) | (IQR) | (IQR) | (IQR) | (IQR) |
| 1 | 6.7 | 3.3 | 24 | 18 | 42 | 43.0 | 273 |
|  | (6.5–7.0) | (3.1–3.4) | (19–35) | (15–21) | (19–60) | (34.0–52.8) | (216–371) |
| 2 | 6.8 | 4.3 | 49 | 26 | 45 | 67.1 | 440 |
|  | (6.5–7.1) | (4.0–4.5) | (43–102) | (21–48) | (32–62) | (46.6–98.1) | (276–700) |
| 3 | 6.0 | 4.0 | 37 | 37 | 50 | 54.9 | 680 |
|  | (5.2–6.3) | (3.4–4.4) | (34–42) | (31–41) | (44–54) | (33.3–64.8) | (209–758) |
| 4 | 5.9 | 4.2 | 41 | 30 | 52 | 56.7 | 423 |
|  | (5.4–6.2) | (3.6–4.3) | (35–47) | (27–39) | (39–66) | (53.7–64.4) | (369–477) |
| 5 | 5.6 | 4.0 | 34 | 37 | 63 | 50.2 | 438 |
|  | (5.5–6.3) | (3.8–4.5) | (25–40) | (35–50) | (50–81) | (38.3–72.7) | (387–689) |
| 6 | 7.0 | 4.3 | 23 | 31 | 51 | 68.2 | 289 |
|  | (6.7–7.2) | (4.2–4.5) | (21–25) | (29–37) | (44–60) | (58.0–76.5) | (266–356) |
| 7 | 5.6 | 4.1 | 46 | 56 | 37 | 91.8 | 361 |
|  | (5.6–6.0) | (4.0–4.2) | (38–50) | (35–82) | (32–43) | (67.5–102) | (330–499) |
| 8 | 7.3 | 4.2 | 14 | 13 | 67 | 43.1 | 683 |
|  | (7.0–7.5) | (4.0–4.5) | (13–16) | (11–15) | (41–92) | (32.1–53.8) | (597–787) |
| 9 | 6.8 | 4.2 | 75 | 71 | 38 | 95.4 | 273 |
|  | (6.4–7.0) | (3.9–4.5) | (62–175) | (27–142) | (37–50) | (84.2–156) | (235–420) |
| median | 6.9 | 4.1 | 27 | 23 | 49 | 52.0 | 420 |
| (IQR) | (6.5–7.2) | (3.5–4.4) | (17–45) | (15–35) | (36–68) | (39.7–71.2) | (274–678) |

Median and interquartile range (IQR) values of total protein (TP), albumin (Alb), AST, ALT, plasma NH₃, glutamate (Glu), and glutamine (Gln) concentrations for each patient in the NO group. The bottom row summarizes the overall group median and IQR.

Supplementary Table S5-B. Laboratory Parameters During the Stable Period in the LO Group

| LO group | | | | | | | |
| --- | --- | --- | --- | --- | --- | --- | --- |
| No | TP (g/dL) | Alb (g/dL) | AST (IU/L) | ALT (IU/L) | NH_3_  (μg/dL) | Glu (μmol/L) | Gln (μmol/L) |
|  | median | median | median | median | median | median | median |
|  | (IQR) | (IQR) | (IQR) | (IQR) | (IQR) | (IQR) | (IQR) |
| 1 | 6.9 | 4.2 | 13 | 9 | 82 | 31.1 | 963 |
|  | (6.8–7.1) | (4.1–4.3) | (13–15) | (8–11) | (69.5–113) | (26.1–38.5) | (873–996) |
| 2 | 7.3 | 4.2 | 21 | 17 | 36 | 48.2 | 711 |
|  | (7.2–7.5) | (4.1–4.4) | (20–27) | (14–21) | (29–54) | (30.6–63.9) | (670–823) |
| 3 | 6.9 | 4.4 | 25 | 14 | 55 | 53.4 | 684 |
|  | (6.8–7.2) | (4.3–4.5) | (23–28) | (13–16) | (38.5–78) | (40.6–71.4) | (661–745) |
| 4 | 6.7 | 4.2 | 33 | 18 | 65 | 60.0 | 785 |
|  | (6.5–7) | (4.1–4.3) | (28–39) | (13–31) | (32–116) | (42.7–81.8) | (633–939) |
| 5 | 6.7 | 3.8 | 15 | 13 | 49 | 47.9 | 708 |
|  | (6.5–6.8) | (3.7–3.9) | (13–16) | (11–16) | (42–64) | (35.5–63.2) | (590–787) |
| 6 | 7.8 | 3.9 | 25 | 46 | 55 | 38.2 | 840 |
|  | (7.6–8.1) | (3.7–4.1) | (21–30) | (38–58) | (40–73) | (32.5–45.3) | (759–939) |
| 7 | 6.7 | 4.0 | 59 | 31 | 44 | 37.3 | 669 |
|  | (6.6–7) | (3.9–4.1) | (48–65) | (20–40) | (38–62) | (30.8–53.8) | (602–709) |
| 8 | 6.8 | 4.0 | 14 | 13 | 83 | 25.3 | 914 |
|  | (6.5–6.9) | (3.9–4.2) | (13–15) | (10–14) | (65–104) | (20.7–34) | (815–955) |
| 9 | 6.7 | 4.2 | 23 | 14 | 66 | 27.9 | 770 |
|  | (6.6–6.9) | (4.1–4.3) | (20–26) | (10–18) | (41–97) | (19–43.9) | (700–1042) |
| Median | 6.9 | 4.1 | 25 | 17 | 55 | 42.7 | 779 |
| (IQR) | (6.7–7.3) | (3.9–4.3) | (18–34) | (12–34) | (39–88) | (30.8–60.0) | (667–909) |

Median and IQR values of TP, Alb, AST, ALT, plasma NH₃, Glu, and Gln concentrations for each patient in the LO group. The bottom row summarizes the overall group median and IQR.
